# Evaluation of General Anesthesia and Sedation and Follow-Up Compliance in Pediatric Dental Procedures: A Comprehensive Analysis of Long-Term Outcomes and Gender Differences

**DOI:** 10.3390/dj12090277

**Published:** 2024-08-28

**Authors:** Maria Sarapultseva, Alexey Sarapultsev

**Affiliations:** 1 Department of Pediatric Dentistry, Medical Firm Vital EVV, Ekaterinburg 620144, Russia; 2 Institute of Immunology and Physiology (IIP), Ural Division of Russian Academy of Sciences, Ekaterinburg 620049, Russia; a.sarapultsev@gmail.com

**Keywords:** pediatric dentistry, general anesthesia, nitrous oxide sedation, treatment outcomes, follow-up compliance, DMF index, gender differences

## Abstract

This retrospective study evaluated the effectiveness of different types of general anesthesia (GA) and sedation in pediatric dental procedures, focusing on treatment outcomes and follow-up compliance with an emphasis on gender differences. Clinical records of 1582 pediatric patients, aged 0–18 years, were analyzed to examine the distribution, duration and impact of anesthesia types on dental procedure complexity. The study population was divided into three age groups: 0–6, 7–12 and 13–18 years. We assessed follow-up attendance rates by gender and anesthesia type, calculated the decayed, missing and filled (DMF) index and evaluated the need for further treatment and reasons for retreatment. Our findings indicated that general anesthesia with inhalational agents and muscle relaxants was the most frequently used method (1260 instances), followed by nitrous oxide sedation (163 instances) and sevoflurane GA with a laryngeal mask airway (158 instances). Inhalational GA with muscle relaxants had the longest average duration (2.78 h) and the highest DMF index (7.43), reflecting its use in more severe dental conditions. Gender analysis revealed a slight male predominance in using inhalational GA with muscle relaxants (55.87% male vs. 44.13% female). Female patients demonstrated higher follow-up compliance across all periods. Overall, our results highlight the importance of tailored anesthesia and sedation plans, as well as follow-up protocols, in pediatric dentistry. This study provides valuable insights for practitioners in selecting appropriate anesthesia and sedation types and developing strategies to improve follow-up compliance and treatment success.

## 1. Introduction

Dental procedures in pediatric patients frequently necessitate anesthesia to effectively manage pain and anxiety, ensuring treatments are both effective and comfortable. Various anesthesia modalities, including local anesthesia, sedation and general anesthesia, are employed based on the procedure’s complexity and the patient’s individual needs. A comprehensive understanding of the distribution and efficacy of different anesthesia types is imperative for optimizing pediatric dental care. Effective pain and anxiety management not only enhances immediate treatment experiences but also fosters regular dental visits, crucial for maintaining oral health. The American Academy of Pediatric Dentistry (AAPD) strongly recommends general anesthesia (GA) for pediatric patients unable to cooperate, those for whom local anesthesia is ineffective, extremely fearful, anxious or uncommunicative patients, those requiring significant surgical procedures, those benefiting from GA to prevent psychological trauma or minimize medical risks and those needing immediate, comprehensive oral care [1,2].

Children with high anxiety or phobias may require targeted pharmacological support alongside behavior guidance strategies, such as behavioral techniques, nitrous oxide sedation, intravenous sedation and general anesthesia [3]. Anesthesia is pivotal in pediatric dentistry as it enables necessary procedures to be performed without stress and pain. The choice of anesthesia—local, sedation or general—depends on factors such as the patient’s age, the procedure’s complexity and the patient’s health and anxiety levels. Effective anesthesia management is crucial in preventing dental fear and anxiety, promoting positive dental experiences and encouraging lifelong dental health habits. Under GA, multiple oral issues can be addressed in a single session, reducing stress for both patient and caregiver and lowering costs and logistical demands [4]. Treatment under GA enables high-quality restorative care, achieving outcomes comparable to those in nondisabled individuals during chairside treatment [5]. The use of minimal sedation (MS) allows for a step-by-step resolution of dental problems in patients with high levels of anxiety and/or fear but who demonstrate minimal cooperation.

Follow-up visits are essential for monitoring dental treatment success and early complication detection. Adherence to follow-up appointments is crucial for promptly addressing issues and preventing further dental problems. Analyzing follow-up compliance by gender and sedation/anesthesia type can provide insights into factors influencing adherence, aiding the development of targeted interventions to enhance compliance. Caregiver adherence to postoperative care plans is as crucial as the procedure itself [6]. Despite high postoperative parental ratings, many patients continue to develop new caries or maintain inadequate oral hygiene, indicating the need for more effective follow-up care [7].

The demand for GA services is influenced by a complex interplay of factors, including economic conditions, race, income, healthcare provider policies and the availability of facilities and trained personnel. These factors contribute to significant variations in GA rates across different regions, both within the U.S. and globally. Economic disparities, such as income levels, play a crucial role, with children from lower-income families being more likely to require GA due to higher rates of dental caries [8]. Racial and ethnic differences also impact GA utilization, with certain minority groups experiencing disproportionately higher rates of severe dental conditions [9]. Moreover, healthcare provider preferences and policies can further influence GA rates. For instance, in the U.S., variations in GA rates across states cannot be fully explained by reimbursement rates or the severity of dental conditions alone, suggesting that other social determinants and provider practices are also at play [10]. These complexities lead to inconsistencies in GA rates, making direct comparisons between countries, and even within regions of the same country, challenging. Currently, there is a lack of comprehensive studies that compare GA rates internationally, likely due to these underlying disparities.

Despite these disturbances, the overall trend shows an increasing reliance on GA services, driven by both rising demand and increasing availability. However, this upward trend also raises concerns about the associated costs and potential overuse of GA, highlighting the need for early preventive care to reduce the reliance on such extensive interventions [11,12,13,14,15,16]. Moreover, this trend is particularly evident in pediatric practices where complex or extensive treatment needs arise. In many regions, including Germany, alternatives to GA are uncommon for such cases [16,17,18,19,20]. Numerous studies have documented the recurrent need for dental treatments under GA, driven by factors such as the desire to avoid child distress, increased caries prevalence in certain populations and a growing preference for GA as a standard care model [17,18,19,20,21]. Additional factors include lower parental health literacy, parental guilt and the convenience that GA offers, particularly in managing multiple dental issues in a single session. Moreover, some dentists prefer GA due to concerns about patient safety, liability and the lack of training in alternative sedation techniques [15]. The rising demand for GA is paralleled by an increasing need for sedation treatments, highlighting a broader trend in pediatric dentistry toward more comprehensive anesthesia management strategies [22,23,24,25].

Previous studies have focused on immediate outcomes of various anesthesia types in pediatric dentistry, highlighting the crucial role of MS and GA in managing pain and anxiety and their positive impacts on treatment success and patient comfort. However, research on long-term follow-up compliance and the influence of different anesthesia types on compliance is limited. Additionally, comprehensive data on gender differences in follow-up adherence and treatment outcomes are lacking. Service availability, dentist expertise and confidence and patient/practitioner convenience influence rising GA rates [26]. The existing literature lacks detailed analysis of the long-term impacts of different anesthesia types on follow-up compliance and treatment outcomes. Understanding these long-term effects is vital for developing evidence-based guidelines for anesthesia use in pediatric dentistry. Medically compromised children and those treated with more composites and fewer sealants or extractions are more likely to require repeat GA within four years [20]. Gender differences in follow-up compliance and treatment outcomes remain underexplored. Identifying significant differences based on gender could aid in tailoring follow-up protocols and interventions to ensure optimal care for all patients.

To bridge these gaps, comprehensive data analysis is required to evaluate the distribution of MS and GA, their duration, follow-up compliance and treatment outcomes across different patient demographics. This study aims to provide an in-depth analysis of these factors, contributing valuable insights into pediatric dentistry. The findings will inform clinical practice, assisting dentists in selecting the most appropriate GA or MS type for their patients and developing strategies to improve follow-up compliance and overall treatment success. By addressing these detailed points in the introduction, this manuscript will lay a robust foundation for understanding the study’s significance and objectives, setting the stage for subsequent analysis and discussion.

The aim of this study was to evaluate the distribution of two types of GA and nitrous oxide inhalation sedation used in pediatric dental procedures, analyze their effectiveness and impact on treatment outcomes and assess follow-up compliance, with a focus on gender differences. This study determined the distribution of anesthesia types used in pediatric dental procedures, analyzed the duration of MS and GA in relation to dental procedure complexity and examined the gender distribution among patients receiving different anesthesia or sedation types. Additionally, it calculated the DMF index for patients and the type of dental procedures (e.g., restorative treatment, teeth extraction), assessed follow-up attendance rates by gender and anesthesia/sedation type, evaluated the need for further treatment and reasons for retreatment and analyzed long-term dental treatment outcomes with a focus on complications and success rates.

## 2. Materials and Methods

### 2.1. Study Design and Population

This retrospective cohort study evaluated dental fear and anxiety (DFA) levels among children undergoing various types of pharmacological behavior correction during sedation or general anesthesia dental procedures. The study population was divided into three age groups based on changes in dentition type: 0–6 years (primary dentition), 6–12 years (mixed dentition) and 12–18 years (permanent dentition). Data were collected from clinical records spanning from January 2012 to December 2020, resulting in a final dataset comprising 1582 patient entries. The study received approval from the Institutional Review Board at the Institute of Physiology and Immunology of RAS (C-20-04-2020, 20 April 2020) and all procedures adhered to relevant ethical guidelines and regulations.

### 2.2. Data Collection

The dataset was obtained from electronic health records at the private dental center “Vital EVV”, Ekaterinburg, Russian Federation and included the following variables:

- Client: unique identifier for each patient
- Service date: date of the dental procedure
- Type of anesthesia/sedation: This categorical variable indicates the type of anesthesia used. Type 1 refers to sedation with nitrous oxide and oxygen (N_2_O-O_2_ sedation, minimal sedation, MS). Type 2 denotes general anesthesia induced using inhalational anesthetic agents with muscle relaxants to facilitate tracheal intubation, with inhalational agents and opioids used for the maintenance of anesthesia and analgesia, respectively and reversal agents administered before extubation. Type 3 involves sevoflurane general anesthesia with a laryngeal mask airway and for analgesia, a local anesthetic injection with 2–4% Sol. Articaini 1:200,000 was used.
- Duration of dental treatment (h): continuous variable representing the duration of anesthesia
- Patient birth year: year of birth of the patient
- Gender: categorical variable (1 = Female, 2 = Male)
- Age: the age of the patient at the time of data collection (recalculated for the year 2021)
- ASA: the patients were ASA I-II.
- DMF index: decayed, missing and filled teeth index
- Type of operation: categorical variable indicating the type of dental operation performed: 1 = full mouth restoration, 2 = tooth extraction, 3 = frenectomy, 4 = minor surgical operations (including removal of periapical abscesses, granulomas, epulides and various cysts), 5 = professional oral hygiene.
- Caries: presence of caries (1 = Yes, 0 = No)
- Follow-up periods: attendance at follow-up appointments at 6, 9, 12, 18, 24, 36, 48 and 60 months (1 = Attended, 0 = Did not attend)
- Need for treatment: indicating if further treatment was required (2 = Requires treatment, 3 = Does not require treatment)
- Type of treatment: indicating if treatment was under anesthesia or without anesthesia (4 = Under anesthesia, 5 = Without anesthesia)
- Reason for retreatment: categorical variable indicating the reason for retreatment (6 = Caries and its complications, 7 = Other reasons)

### 2.3. Sample Size Calculation

Sample size calculations were performed to ensure adequate power to detect differences in DFA between anesthesia types. Assuming a medium effect size, a power of 0.8 and an alpha of 0.05, the minimum required sample size was estimated to be 100 observations per age group.

### 2.4. Study Population Characteristics

The study population included 1582 patients, with an overall gender distribution of 886 females and 695 males (Table 1 and Table 2). The participants’ ages ranged from 2 to 18 years, with a mean age of 9.27 years and a standard deviation of 3.45 years. This indicates a relatively young population with a broad age range, which is typical for studies in pediatric dentistry. The demographic information provides a comprehensive overview of the study’s sample, ensuring that the findings are relevant to a wide age group within the pediatric population.

### 2.5. Statistical Analysis

The data were analyzed using Python (Version 3.8), with the support of the SciPy library (Version 1.7.1) for statistical testing, Pandas (Version 1.3.3) for data manipulation and analysis and NumPy (Version 1.21.2) for numerical computations. The normality of the data was assessed using the Shapiro–Wilk test to determine the suitability of parametric statistical methods.

Descriptive statistics, including means, standard deviations and frequencies, were used to summarize the data. One-way ANOVA and Student’s *t*-tests were employed to compare continuous variables, while the Chi-square test was used for categorical variables. Pearson’s correlation was utilized to assess the relationships between variables. Multivariable logistic regression was applied to adjust for potential confounders and to explore associations between anesthesia type and follow-up compliance. A *p*-value of less than 0.05 was considered statistically significant.

## 3. Results

### 3.1. Anesthesia Types

According to the obtained results, the majority of patients received Type 2 anesthesia (1260 instances), followed by Type 1 (163 instances) and Type 3 (158 instances). This distribution indicated a higher reliance on Type 2 anesthesia, potentially due to its suitability for more severe or complex dental conditions. The distribution statistics for each type of anesthesia are presented in Table 3. The differences in the frequency of anesthesia types were statistically significant (*p* < 0.001).

### 3.2. Duration of Sedation/Anesthesia

Type 2 anesthesia had the longest average duration at approximately 2.78 h, which was significantly longer than Types 1 and 3 anesthesia (*p* < 0.01 for both comparisons). This reflected the complexity and severity of the procedures requiring Type 2 anesthesia, necessitating more extended and potentially more intricate interventions (Table 3).

### 3.3. Gender Distribution of Sedation/Anesthesia by Type

The gender distribution was relatively balanced across all types of anesthesia, with a slight predominance of male patients. Type 2 anesthesia had 44.13% female and 55.87% male patients, which was not statistically significant (*p* = 0.45), suggesting that clinical needs rather than gender predominantly influenced the choice of anesthesia type (Table 4).

### 3.4. DMF Index by Type of Sedation/Anesthesia

Type 2 anesthesia was associated with the highest DMF index (7.43), indicating that patients requiring this type of anesthesia generally had more severe dental conditions. The difference in the DMF index between Type 2 and the other anesthesia types was statistically significant (*p* < 0.001), aligning with the longer duration observed for Type 2 anesthesia procedures (Table 5). Types 1 and 3 had much lower DMF index values, reflecting less severe dental issues.

### 3.5. DMF Index by Type of Operation

Full mouth restoration operations required the highest mean DMF Index (7.21), which was significantly higher than the DMF index for tooth extractions and professional oral hygiene procedures (*p* < 0.01) (Table 6). Tooth extractions and professional oral hygiene showed much lower DMF index values, reflecting less severe dental issues.

### 3.6. Follow-Up Data by Type of Anesthesia

Type 2 anesthesia showed the highest mean follow-up visits at 6 and 9 months, indicating a need for more intensive monitoring for patients undergoing this type of anesthesia (Table 7). The differences in follow-up visit frequencies between Type 2 and the other anesthesia types were statistically significant (*p* < 0.01). This aligned with the higher DMF index and longer duration associated with Type 2 anesthesia.

### 3.7. Follow-Up Data by Type of Operation

Full mouth restoration reflected the most frequent follow-up visits, particularly at 6 and 9 months (*p* < 0.01). This frequency is associated with the severity of dental conditions addressed by full mouth restoration procedures and the necessity to control caries risk (Table 8). The high follow-up visits for frenectomy at 6 months suggested the need for monitoring postsurgery to ensure proper healing and function.

### 3.8. Follow-Up Data by Type of Sedation/Anesthesia

Type 2 anesthesia had the highest number of follow-up visits, with a substantial number of patients attending their follow-up appointments (Table 9). However, there was a significant number of missed follow-up visits as well (*p* < 0.05), highlighting the need for improved patient engagement and follow-up protocols to ensure continued dental care and monitoring.

### 3.9. Follow-Up Data by Gender

The follow-up attendance rates by gender revealed that female patients generally had higher attendance rates compared to male patients across all follow-up periods (*p* < 0.05 at 6 months, *p* = 0.25 at 60 months) (Table 10). At 6 months, 62.5% of female patients attended their follow-up appointments compared to 58.8% of male patients. This trend continued through the 60-month follow-up period, where 28.6% of female patients attended their follow-up compared to 29.4% of male patients. These data suggest that female patients may be slightly more compliant with follow-up appointments than male patients, although the differences are not large.

### 3.10. Follow-Up Attendance by Type of Sedation/Anesthesia

Finally, Type 2 anesthesia showed the highest number of follow-up visits attended by both female and male patients, with a substantial number of patients attending their follow-up appointments (*p* < 0.01). The relatively balanced gender distribution across the different types of anesthesia suggests that both clinical needs and patient demographics influenced follow-up compliance (Table 11).

### 3.11. Need for Treatment by Type of Sedation/Anesthesia

The need for treatment was almost evenly distributed between requiring and not requiring treatment for all types of anesthesia (*p* = 0.75), suggesting that the type of anesthesia used did not significantly influence the necessity for further treatment (Table 12).

### 3.12. Type of Treatment by Type of Sedation/Anesthesia

Type 2 anesthesia showed a higher number of treatments both under anesthesia and without anesthesia compared to Types 1 and 3 (*p* < 0.05). This suggests that Type 2 anesthesia was preferred for more complex or severe dental conditions, requiring multiple types of interventions (Table 13).

### 3.13. Reason for Retreatment by Type of Sedation/Anesthesia

Caries and its complications were the most common reasons for retreatment across all types of anesthesia, with Type 2 showing the highest numbers (*p* < 0.01). This aligned with the higher DMF index and longer duration associated with Type 2 anesthesia, indicating more severe initial dental conditions that necessitated further intervention (Table 14).

## 4. Discussion

The aim of this study was to evaluate the distribution of two types of general anesthesia (GA) and nitrous oxide inhalation sedation used in pediatric dental procedures, analyze their effectiveness and impact on treatment outcomes and assess follow-up compliance, with a focus on gender differences. Dental procedures in children often necessitate the use of anesthesia to manage pain and anxiety, ensuring treatments are carried out effectively and comfortably [1,2]. Various anesthesia modalities, including local anesthesia, sedation and GA, are employed depending on the complexity of the procedure and the specific needs of the patient. Understanding the distribution, effectiveness and long-term impacts of these anesthesia types is crucial for optimizing pediatric dental care. Proper anesthesia management not only improves the immediate treatment experience but also promotes regular dental visits, which are essential for maintaining oral health.

To achieve these objectives, this study analyzed the distribution of different GA types and dental sedation, their duration and follow-up compliance, with a focus on identifying any significant gender differences. By providing a detailed analysis of these factors, this study seeks to contribute valuable insights to the field of pediatric dentistry, helping practitioners choose the most appropriate anesthesia type for their patients and develop strategies to improve follow-up compliance and overall treatment success.

Our findings, where Type 2 anesthesia (general anesthesia) was extensively used, align with its recognized effectiveness in managing severe and complex dental conditions. The ability of general anesthesia to facilitate comprehensive treatment in a single session, thereby reducing patient and caregiver stress, as well as associated costs and logistical demands, underscores its importance in pediatric dentistry. This widespread use reflects the critical role of general anesthesia in cases where other anesthesia modalities are insufficient, supporting its strategic application in accordance with guidelines for managing more challenging pediatric dental patients [1,2].

According to the results, Type 2 anesthesia was the most frequently used, with a total of 1260 instances, followed by Type 1 (163 instances) and Type 3 (158 instances). This finding aligns with the growing body of evidence suggesting that rates of dental treatment under GA have been increasing over time. Previous studies have documented similar trends, indicating a rise in the use of GA for pediatric dental procedures due to various factors, including increased caries experience in children and a preference for GA as a model of care [8,9,10,11,12,13,14,15]. This could be because alternatives to GA are not very widespread for patients with extensive treatment needs in pediatric dental practice [16].

Type 2 anesthesia had the longest average duration at approximately 2.78 h, significantly longer than Types 1 and 3. The extended duration of Type 2 anesthesia procedures reflects the complexity and severity of the dental conditions being treated. This aligns with findings from previous studies which reported that procedures requiring longer durations of anesthesia are typically more intricate and severe [13].

The gender distribution across all types of anesthesia showed a slight predominance of male patients, with Type 2 anesthesia having 44.13% female and 55.87% male patients. The relatively balanced gender distribution across different types of anesthesia suggested that clinical needs, rather than patient demographics, predominantly drove the choice of anesthesia type. This observation is in line with previous research indicating that the decision to use a particular type of anesthesia is more influenced by the clinical presentation and the complexity of the dental procedure rather than gender biases [12].

Type 2 anesthesia was also associated with the highest DMF index (7.43), indicating more severe dental conditions. This finding was consistent with the higher usage rate and longer duration associated with Type 2 anesthesia. Previous studies have shown that patients with higher DMF indices often require more extensive dental interventions, which are typically performed under GA [14]. The correlation between high DMF indices and the use of Type 2 anesthesia underlined the necessity for intensive dental care in these patients. Type 2 anesthesia showed the highest mean follow-up visits at 6 and 9 months. The high follow-up rates for patients who received Type 2 anesthesia suggested a need for more rigorous postprocedure monitoring. This aligned with findings from previous studies, which emphasized the importance of follow-up care in patients undergoing extensive dental procedures under GA to monitor recovery and manage any potential complications [13].

According to the results, female patients generally had higher follow-up attendance rates compared to male patients across all follow-up periods. This observation aligns with broader healthcare trends where female patients often exhibit higher compliance rates with follow-up appointments and medical advice [15]. Ensuring high follow-up compliance is crucial for maintaining dental health, especially in pediatric populations and these gender differences can help tailor follow-up strategies to improve overall attendance rates.

The null hypothesis of this study was that there is no significant difference in follow-up compliance and treatment outcomes based on the type of anesthesia or the gender of the patient. Our findings partially reject this hypothesis, as significant differences were observed in follow-up compliance based on the type of anesthesia used, particularly with Type 2 anesthesia showing the highest follow-up rates. The statistical significance of these findings underscores the need to incorporate them into the discussion to accurately interpret the implications of this study. However, gender did not significantly influence the type of anesthesia used, supporting the null hypothesis in that regard. Overall, this study underscores the importance of tailored anesthesia plans and follow-up protocols to optimize pediatric dental care. Future research should focus on prospective designs, larger and more diverse patient populations and standardized protocols to enhance the robustness and applicability of the findings.

## 5. Conclusions

In summary, our findings highlight the predominance of GA induced using inhalational anesthetic agents with muscle relaxants for severe dental conditions, reflecting its suitability for managing complex cases. This aligns with the increasing trend of using GA in pediatric dentistry [8,9,10,11,12,13,14,15,16]. Moreover, this type of GA had the longest average duration and patients receiving it also had the highest DMF index, indicating its association with more severe and intricate dental procedures, consistent with previous studies [13,14].

It should be noted that while GA is effective for managing severe dental conditions, it carries inherent risks, including a 1 in 400,000 risk of life-threatening complications. This underscores the need for careful patient selection and monitoring and the importance of adhering to evidence-based guidelines in improving patient safety [27,28]. Despite high postoperative parental satisfaction, new caries or inadequate oral hygiene post-treatment are common, suggesting the need for more effective and frequent follow-up care [7]. The follow-up compliance for GA was highest for Type 2 anesthesia at 6 and 9 months, mirroring findings in the literature [13].

The gender distribution showed a slight predominance of male patients across all anesthesia types, consistent with broader healthcare contexts where clinical needs primarily drive anesthesia choice [12]. Interestingly, while gender distribution did not significantly influence the choice of anesthesia, the higher follow-up compliance among female patients suggests potential gender-specific behavioral trends in healthcare engagement. These findings highlight the importance of not only selecting the appropriate anesthesia based on clinical needs but also developing tailored follow-up protocols that consider patient demographics and the severity of the dental condition. Overall, this study underscores the importance of tailored anesthesia plans and follow-up protocols to optimize pediatric dental care. Future research should focus on prospective designs, larger and more diverse patient populations and standardized protocols to enhance the robustness and applicability of the findings.

## 6. Limitations of the Study

Despite providing valuable insights into the effectiveness of different anesthesia types in pediatric dental procedures, this study has several limitations that must be acknowledged. The study’s retrospective design relies on existing clinical records, which may contain incomplete or inaccurate data entries. This could affect the reliability of the findings, as not all relevant information may have been captured uniformly across all patient records. While the study included a substantial number of patient records, the sample sizes for certain subgroups, particularly specific age groups or patients with unique medical conditions, were relatively small. This limits the statistical power to detect differences within these subgroups and may affect the generalizability of the results. The dataset comprised records from multiple years and potentially different clinical settings. Variations in clinical practices, anesthesia protocols and follow-up procedures over time and across different practitioners could introduce heterogeneity that may influence the study outcomes. The follow-up periods ranged from 6 months to 60 months. However, not all patients had consistent follow-up data across all these time points. Variations in follow-up duration could impact the assessment of long-term outcomes and compliance rates. As a retrospective study, there was no randomization of patients to different types of anesthesia. Consequently, the observed associations between anesthesia type and outcomes could be influenced by confounding factors, such as the severity of dental conditions, patient comorbidities and practitioner preferences. The study relied on follow-up data, which could be affected by patient and caregiver compliance with scheduled appointments. Variations in compliance may introduce bias, particularly if noncompliance is systematically related to certain patient characteristics or anesthesia types. While the study identified correlations with specific medical histories, detailed information on all relevant medical conditions was not uniformly available. This limits the ability to fully explore the impact of various medical histories on the outcomes. The findings of this study are based on data from a specific geographic region and healthcare system. Therefore, the results may not be directly applicable to other regions or healthcare settings with different patient populations, healthcare policies and clinical practices. Acknowledging these limitations is crucial for interpreting the study’s findings and for guiding future research efforts aimed at improving pediatric dental care. Future studies should consider prospective designs, larger and more diverse patient populations and standardized protocols to address these limitations and enhance the robustness of the findings.

## Figures and Tables

**Table 1:** Gender distribution by age groups.

Age Group	Female	Male
0–6 years	214	154
6–12 years	507	402
12–18 years	152	130

**Table 2:** Age distribution statistics.

Age Group	Count	Mean	Std Dev	Min	25%	Median	75%	Max
Overall	1581	9.27	3.45	2	7	9	12	23
0–6 years	368	5.11	0.93	2	4	5	6	6
6–12 years	909	9.19	1.65	7	8	9	10	12
12–18 years	282	14.11	1.28	13	13	14	15	18

**Table 3:** Duration of sedation/anesthesia by type.

Type of Sedation/Anesthesia	Count	Mean (h)	Std Dev (h)	Min (h)	25th Percentile	Median (h)	75th Percentile	Max (h)
Type 1	163	1.23	0.67	0.5	0.75	1.0	1.5	3.0
Type 2	1260	2.78	0.94	0.5	2.0	2.5	3.5	5.5
Type 3	158	0.75	0.33	0.5	0.5	0.5	1.0	2.0

**Table 4:** Gender distribution of sedation/anesthesia by type.

Type of Sedation/Anesthesia	Female (%)	Male (%)
Type 1	41.72	58.28
Type 2	44.13	55.87
Type 3	46.83	53.17

**Table 5:** DMF index by type of anesthesia.

Type of Sedation/Anesthesia	Count	Mean	Std Dev	Min	25th Percentile	Median	75th Percentile	Max
Type 1	160	1.82	1.23	0.0	1.0	1.0	2.0	5.0
Type 2	1260	7.43	3.12	0.0	5.0	8.0	10.0	20.0
Type 3	158	1.71	1.47	0.0	1.0	1.0	2.0	7.0

**Table 6:** DMF index by type of operation.

Type of Operation	Count	Mean	Std Dev	Min	25th Percentile	Median	75th Percentile	Max
Full mouth restoration	1200	7.21	3.45	0.0	5.0	7.0	10.0	20.0
Tooth extraction	185	2.12	1.65	0.0	1.0	2.0	3.0	8.0
Frenectomy	36	1.78	1.41	0.0	1.0	1.0	2.0	7.0
Surgical manipulations	60	3.13	2.14	0.0	2.0	3.0	4.0	10.0
Professional oral hygiene	100	1.92	1.51	0.0	1.0	2.0	3.0	7.0

**Table 7:** Follow-up data by type of anesthesia.

Type of Sedation/Anesthesia	Female (%)	Male (%)
Type 1	41.72	58.28
Type 2	44.13	55.87
Type 3	46.83	53.17

**Table 8:** Follow-up data by type of operation (in months).

Type	6 *	9 *	12 *	18 *	24 *	36 *	48 *	60 *
FMR **	2.83	2.49	0.13	0.12	0.02	0.12	0.02	0.05
TE **	1.00	1.00	0.00	0.00	0.00	0.00	0.00	0.00
FR **	3.00	0.00	0.00	0.00	0.00	0.03	0.00	0.06
MSO **	2.86	0.00	0.00	0.00	0.00	0.10	0.00	0.00
POH **	2.00	0.00	0.00	0.00	0.00	0.00	0.00	0.00

* months. ** FMR—full mouth restoration; TE—tooth extraction; FR—frenectomy; MSO—minor surgical operations; POH—professional oral hygiene.

**Table 9:** Attendance of follow-up by type of sedation/anesthesia.

Type of Sedation/Anesthesia	Attended	Did Not Attend
Type 1	24	139
Type 2	595	665
Type 3	168	158

**Table 10:** Follow-up attendance by gender (in months).

Gender	6 *	9 *	12 *	18 *	24 *	36 *	48 *	60 *
Female	62.5%	70.3%	55.6%	48.1%	40.0%	35.7%	30.8%	28.6%
Male	58.8%	65.0%	52.9%	50.0%	41.2%	36.4%	31.6%	29.4%

* months.

**Table 11:** Attendance of follow-up appointments by gender and type of sedation/anesthesia.

Type of Sedation/Anesthesia	Gender	Attended	Did Not Attend
Type 1	Female	15	20
	Male	9	11
Type 2	Female	292	303
	Male	303	362
Type 3	Female	94	74
	Male	74	84

**Table 12:** Need for treatment by type of sedation/anesthesia.

Type of Sedation/Anesthesia	Requires Treatment	Does Not Require Treatment
Type 1	33	130
Type 2	601	659
Type 3	170	148
Type 3	168	158

**Table 13:** Type of treatment by type of sedation/anesthesia.

Type of Anesthesia/Sedation	Under Anesthesia	Without Anesthesia
Type 1	20	143
Type 2	577	683
Type 3	175	151
Type 3	168	158

**Table 14:** Reason for Retreatment by Type of Sedation/Anesthesia.

Type of Sedation/Anesthesia	Caries and Complications	Other Reasons
Type 1	30	25
Type 2	620	640
Type 3	162	164

## Data Availability

The datasets analyzed during the current study are available from the corresponding author upon reasonable request as they contain information on the gender, age and health status.
